# A Replicable and Reproducible Digital Method for Quantifying Maxillary Sinus Airway Changes after Sinus Lifts Using the Lateral Window Approach Technique—A Retrospective Study

**DOI:** 10.3390/jpm11111093

**Published:** 2021-10-26

**Authors:** Héctor González Menéndez, Paulina Rodríguez Torres, Blanca Muñoz Jiménez, Agustín Galparsoro Catalán, Pilar Velasco Bohórquez, Georgia Tzironi, Lara San Hipólito Marín, Álvaro Zubizarreta Macho, Sofía Hernández Montero

**Affiliations:** 1 Department of Implant Surgery, Faculty of Health Sciences, Alfonso X el Sabio University, 28691 Madrid, Spain; hgonzmen@uax.es (H.G.M.); prodrtor@uax.es (P.R.T.); bmunojim@myuax.com (B.M.J.); agalpcat@uax.es (A.G.C.); mvelaboh@uax.es (P.V.B.); lsanhmar@uax.es (L.S.H.M.); shernmon@uax.es (S.H.M.); 2 Department of Surgery, Faculty of Medicine, University of Salamanca, 37008 Salamanca, Spain; gtzironi@mail.com

**Keywords:** maxillary sinus, sinus lift, airway volume, cone beam computed tomography scan, lateral window approach

## Abstract

In the present retrospective study, we aimed to assess the replicability and reproducibility of a novel digital measurement technique for analyzing the volumes of the left and right maxillary sinuses and the nasal and maxillary sinus airway complex after a sinus lift procedure using the lateral window approach, to provide an accurate measurement technique for easily applying in clinical practice and to allow pre-operative assessment of maxillary sinus lift surgery, avoiding complications and making surgery more predictable. Material and Methods: Thirty patients with partially edentulous posterior maxilla were selected and submitted to bilateral sinus lift using the lateral window approach technique, with grafting materials selected and submitted to cone beam computed tomography (CBCT) scans, both pre- and postoperatively. Then, datasets were uploaded to therapeutic digital planning software to measure the volume of the right and left maxillary sinuses and the nasal and maxillary sinus airway complex. Gage R&R statistical analysis was performed to assess the replicability and reproducibility of the digital measurement technique. Results: The variability attributable to the novel digital measurement technique was 3.4% for replicability and 3.4% for reproducibility of the total variability of the samples. Conclusion: The novel digital method proposed is a replicable and reproducible technique for analyzing the volume of the right and left maxillary sinuses and the nasal and maxillary sinus airway complex after a sinus lift using the lateral window approach technique, allowing an accurate pre-operative assessment of maxillary sinus lift surgery, avoiding complications and making surgery more predictable.

## 1. Introduction

The first upper molar has the highest rates of cavities and periodontal disease, followed by the third upper molar and upper premolars. These conditions can lead to premature tooth loss [1]. Dental extractions can cause the bone tissues to undergo volumetric changes characterized by the resorption of the alveolar process and the pneumatization of the maxillary sinus—particularly in the upper jaw, due to the centripetal bone resorption pattern—decreasing bone availability and complicating the rehabilitation of edentulous patients using dental implants [2]. In addition, the presence of the maxillary sinus in the posterior upper maxilla limits bone availability and thus inhibits dental implant placement for rehabilitation of posterior edentulism [3]. Therefore, bone augmentation procedures are an option for increasing bone availability and enabling the placement of dental implants, including grafting procedures, apposition grafts (with or without Le Fort I osteotomies), short dental implants, and sinus lifts. Additionally, sinus lifts involve a bone augmentation procedure that necessitates a maxillary sinus approach, which is the largest cavity of the paranasal sinuses with average measurements of 40 mm height, 20 mm length, 30 mm depth [4], and a mean volume of 11.3 ± 4.60 cm^3^ [5].

A sinus lift using the lateral window approach technique was first noted by Tatum (1977) [6] and subsequently developed by Boyne and James (1980) to enable access to the maxillary sinus through a lateral window, preserving the Schneider membrane and filling the space between the maxillary sinus floor and the Schneider membrane with autologous bone graft or biomaterials [7]. This surgical procedure requires a preoperative assessment of the bone augmentation needed for dental implant placement that will provide the grafting material volume [8]. Therefore, radiographic techniques such as orthopantomography, Waters’ projections, lateral skull radiography, and Caldwell projection [9,10,11] have been used to plan the bone augmentation procedure. However, two-dimensional radiographs provide limited information compared with advanced radiographic techniques such as computerized axial tomography and cone beam computerized tomography (CBCT), which provide complete three-dimensional information in all planes [9]. Additionally, the airway volume of the nasal and maxillary sinus has been previously analyzed using lineal measurement procedures [10], mathematical equations [11], lateral and anteroposterior radiographs [12,13,14,15,16], computer tomography (CT) [17], and acoustic rhinometry [18,19,20,21], but some of these measurement procedures do not enable accurate measuring of the total volume of the nasal and maxillary sinus or have a steep learning curve. As a result, digital measurement methods have been proposed for analyzing the volume of anatomical structures [22,23,24], although they have yet to be properly assessed.

The size of the maxillary sinus can be affected by infection, injury, irradiation, or syndromes during the development process (12–15 years) [25], leading to a hypoplasia in 1.5–10% of the population [24], which can cause headaches, facial pain, and nasal symptoms [26]; moreover, post-sinus surgery has been also highlighted as an etiologic factor that can influence the hypoplasia of the maxillary sinus [27]; therefore, pre-operative diagnosis of altered anatomy in the sinonasal complex is crucial in dental implant surgery or sinus surgery [28]. Hence, an accurate, repeatable, and reproducible measurement technique for analyzing pre-operatively the volume of the nasal and maxillary sinuses airway complex would be useful for accurately planning the surgical treatment approach. Furthermore, the mucosal thickening (OR 5.2, 95% CI 2.0–17.3), and anatomical variations in the sinonasal complex, such as the deviated/hyperplastic meatus (OR 1.6, 95% CI 1.4–2.1) (48.8%), have been highly associated to the hypoplasia of the maxillary sinus [28]; although Karslioglu H and Sumer AP did not show statistically significant correlation between implant applications and sinus pathologies with both internal and external elevation procedures and implant applications (*p* > 0.05) [29]. Additionally, the decrease in the volume of the maxillary sinus has been theorized as a potential predisposing factor for the development of obstructive sleep apnea, which is defined as a type of sleep apnea caused by partial or complete obstruction of the upper pharyngeal airway, preventing normal breathing during sleep [30]. Kim et al. showed a significant (*p* = 0.029) association between obstructive sleep apnea and the decreased ratio of maxillary sinus volume to the whole nasal airway in adults, concluding that the interventions that decrease the maxillary sinus volume, such as maxillary dental implants with sinus lift procedures, might contribute to the development of obstructive sleep apnea [31]. This necessitates accurate measuring of the volume changes of the maxillary sinus after sinus lift using the lateral window approach technique.

In addition, the clinical interest of this study is to provide the implantologist with an accurate measurement technique for pre-operative assessment of maxillary sinus lift surgery, avoiding complications and making surgery more predictable.

The aim of this study is to assess the replicability and reproducibility of an innovative digital measurement technique for analyzing the volume of the left and right maxillary sinuses and the nasal and maxillary sinus airway complex after a sinus lift using the lateral window approach technique, with a null hypothesis (H_0_) that the novel method will not provide replicable and reproducible volume measurements.

## 2. Experimental Section

### 2.1. Study Design

A retrospective study was carried out at the Department of Implant Surgery at Alfonso X el Sabio University (Madrid, Spain) between November 2020 and February 2021. The study was approved by the Ethical Committee of the Faculty of Dentistry, Alfonso X el Sabio University (Madrid, Spain) in October 2020 (process No. 16/2020). Patients were treated at the Dental Centre of Innovation and Advanced Specialties at Alfonso X El Sabio University (Madrid, Spain) between June 2016 and March 2019 for the implant-supported rehabilitation of partially edentulous posterior maxilla via sinus lift using the lateral window approach technique with analogous grafting materials. The patients gave their consent to provide preoperative and postoperative CBCT scans.

### 2.2. Clinical Procedure

Thirty patients (15 men and 15 women) between 67 and 72 years old with partially edentulous posterior maxilla were selected and underwent a bilateral sinus lift using the lateral window approach technique with analogous grafting materials. The inclusion criteria were adult patients with no history of systemic conditions and those who experienced a perforated Schneider membrane during the sinus lift procedure. Excluded from this study were patients with osteoporosis, neoplasia, acute maxillary sinusitis, acute oral infections, coagulation disorders, or a history of chemotherapy or radiotherapy in the area of the head or neck, as well as immunocompromised patients, those undergoing bisphosphonate therapy, smokers (10 or more cigarettes per day), and patients with chronic alcohol or drug abuse issues.

**Figure 1:**
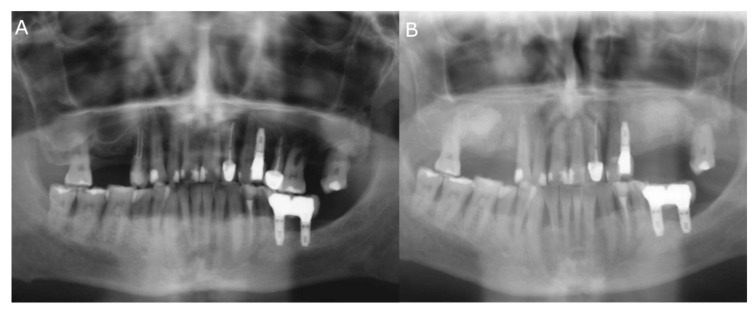
(**A**) Preoperative and (**B**) postoperative orthopantomography radiography after bilateral sinus lift using the lateral window approach technique.

Sinus lift procedures were performed following infiltrative anesthesia using 2% lidocaine and 1:100,000 epinephrine (Artinibsa; Inibsa, Lliça de Vall, Barcelona, Spain). Subsequently, a full-thickness flap was lifted to enable osteotomy preparation with a piezoelectric device. Afterwards, a 3.2 mm osteotome was used to elevate the Schneiderian membrane. The Valsalva maneuver test was performed to assess whether or not the sinus membrane remained intact after the osteotome procedure. Finally, the grafting material was placed under the previously lifted Schneiderian membrane (Figure 1). All the regeneration procedures were performed by the same group of surgeons from the Master Degree of Dental Implants and Implant-supported Prostheses program at Alfonso X el Sabio University (Madrid, Spain).

### 2.3. Measurement Procedure

**Figure 2:**
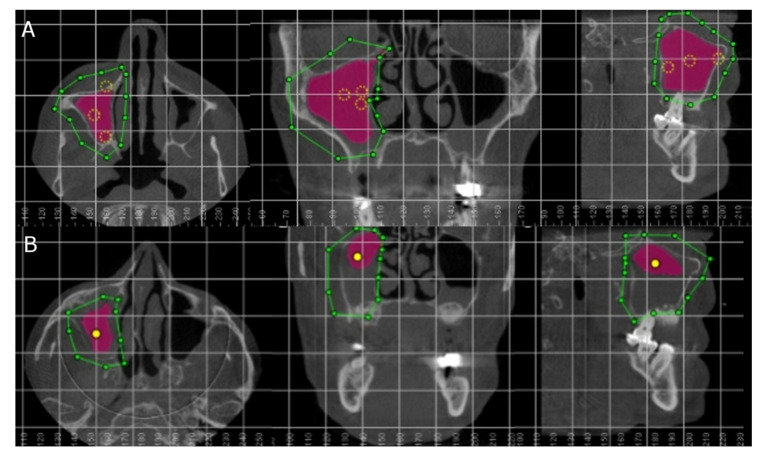
(**A**) Axial, coronal, and sagittal plane of the preoperative and (**B**) postoperative CBCT scans. Green line describes the selected area, yellow points define the air density, and purple area indicate the volume airway of the right maxillary sinus.

All patients underwent preoperative (Figure 2A) and postoperative (Figure 2B) cone beam computed tomography (CBCT) scans (WhiteFox, Satelec, Merignac, France), for sinus lift and dental implant placement planning, 8 months after the sinus lift using the lateral window approach technique under the following exposure parameters: 105.0 kV peak, 8.0 mA, 7.20 s, and 15 mm × 13 mm field of view, aligning the Frankfort plane to the floor with frontal and chin support. Afterwards, the preoperative and postoperative CBCT scans (WhiteFox, Satelec, Merignac, France) were uploaded to therapeutic digital planning software (Dolphin Imaging, Dolphin Imaging & Management Solutions, Chatsworth, CA, USA) for accurate measurement of the volume of the left maxillary sinus, right maxillary sinus, and the nasal and maxillary sinus airway complex. The airway volumes were measured after selecting the anatomical area in the axial, coronal, and sagittal plane, ensuring accurate air density measurement by placing reference points inside the selected area. Afterwards, a tissue density with a tolerance range of ±500 Hounsfield units (HU) was selected according to the air density.

**Figure 3:**
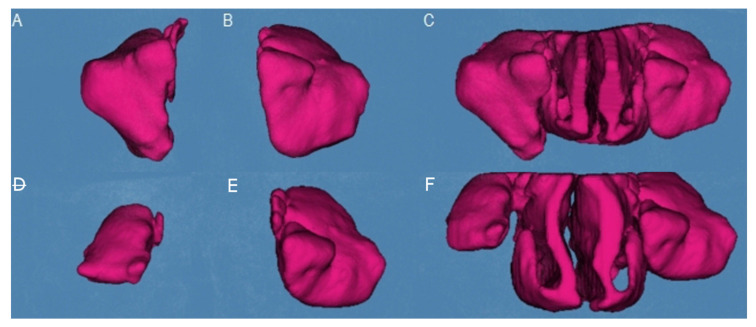
(**A**) Preoperative volumetric assessment of the right maxillary sinus, (**B**) left maxillary sinus, and (**C**) nasal and maxillary sinus airway complex. (**D**) Postoperative volumetric assessment of the right maxillary sinus, (**E**) left maxillary sinus, and (**F**) nasal and maxillary sinus airway complex.

Subsequently, therapeutic digital planning software (Dolphin Imaging, Dolphin Imaging & Management Solutions, Chatsworth, CA, USA) was used for the accurate measurement of the volume of the right maxillary sinus (Figure 3A), left maxillary sinus (Figure 3B), and nasal and maxillary sinus airway complex (Figure 3C) after palatine expansion using the Airway Measurement tool.

**Figure 4:**
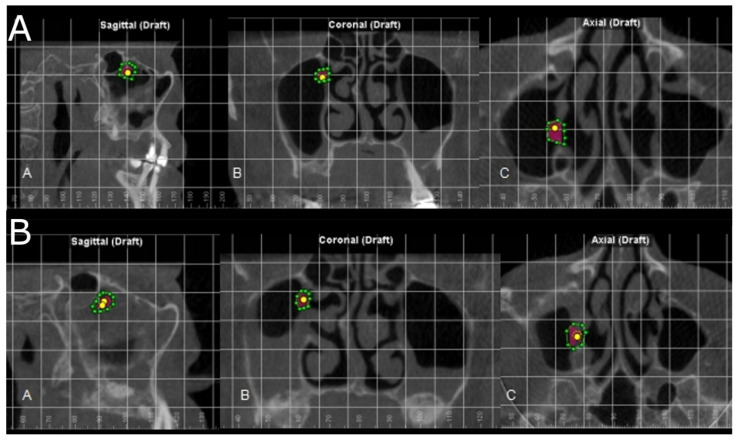
(**A**) Preoperative and (**B**) postoperative assessment of the position and permeability of the maxillary sinus ostium in the axial, coronal, and sagittal plane.

In addition, the position and permeability of the maxillary sinus ostium were also analyzed before and after sinus lifts using the novel digital method proposed, in order to assess the prevalence of maxillary sinus stenosis and, hence, an increased risk of sinus lift complications and obstructive sleep apnea [32] (Figure 4).

### 2.4. Confirmation of Replicability and Reproducibility of the Technique

In order to confirm the replicability and reproducibility of this digital measurement technique, cases were randomly (Epidat 4.1, Galicia, Spain) selected and measured two times by two operators (Operators A and B). A Gage R&R statistical analysis was performed.

### 2.5. Statistical Tests

Statistical analysis was carried out using SAS v9.4 (SAS Institute Inc., Cary, NC, USA) and R (R Foundation for Statistical Computing, Vienna, Austria). Means and standard deviation (SD) values were used for the descriptive statistical analysis of quantitative variables. The replicability and reproducibility of this digital measurement technique were assessed using Gage R&R statistical analysis. Statistical significance was defined as *p* < 0.05.

## 3. Results

Table 1 shows the mean and SD values for the preoperative and postoperative volumes of the left maxillary sinus (mm^3^), right maxillary sinus (mm^3^), and nasal and maxillary sinus airway complex (mm^3^) after sinus lift using the lateral window approach technique. Volume differences of the left maxillary sinus (mm^3^), right maxillary sinus (mm^3^), and nasal and maxillary sinus airway complex (mm^3^) after sinus lift using the lateral window approach technique are also shown in Table 1.

Table 2 shows the mean and SD values for the two measurements performed by the two operators for the Gage R&R statistical analysis.

**Figure 5:**
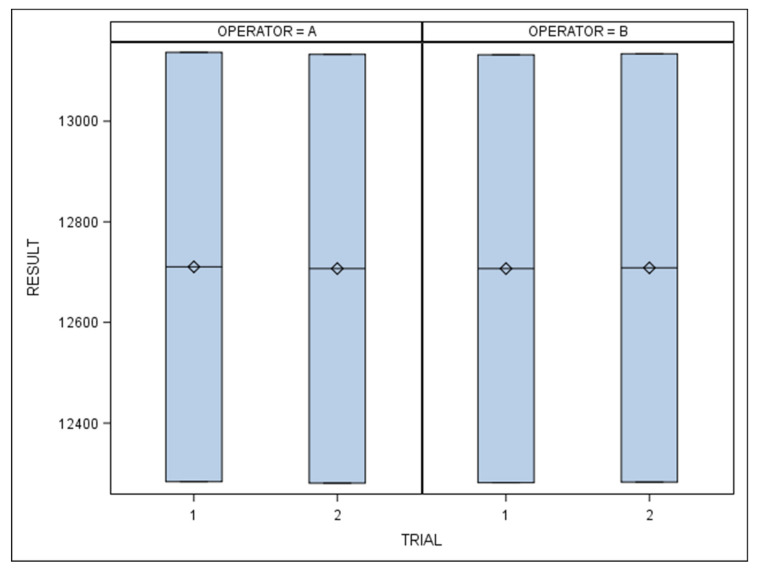
Box plots of results obtained by each operator in each trial. The horizontal lines represent each box’s respective median value. ◇: mean value of the box plots.

The Gage R&R statistical analysis of the proposed digital measurement technique found no statistically significant differences (*p* = 0.478) (Figure 5).

**Figure 6:**
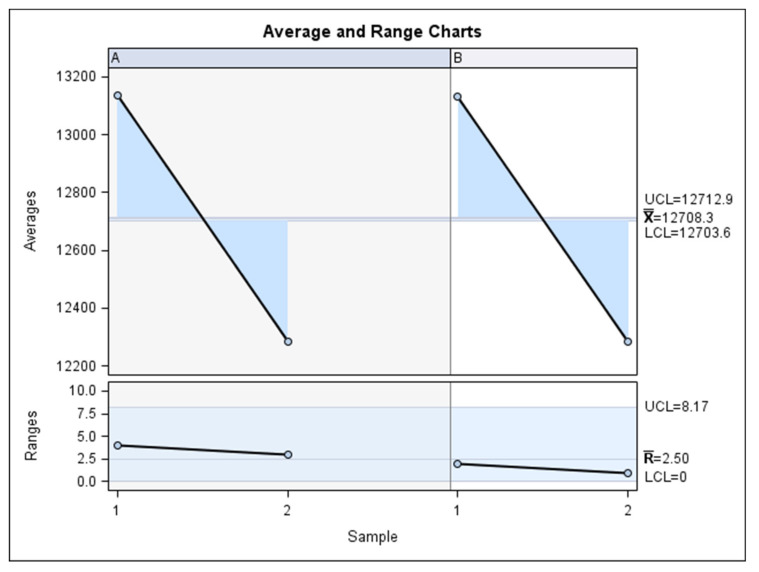
Charts for the average of the measures of the volume of the right maxillary sinus, left maxillary sinus, and the nasal and maxillary sinus airway complex as assessed by two operators.

**Figure 7:**
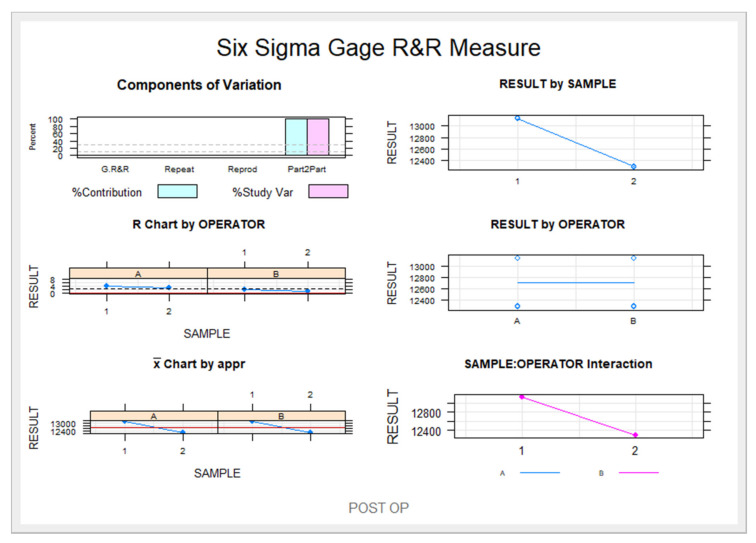
Measurement system analysis related to the volume of the right maxillary sinus, left maxillary sinus, and the nasal and maxillary sinus airway complex with a chart showing the influence of each component on the total variance (Components of Variation), a mean control chart, a range control chart (R Chart by OPERATOR and x Chart by appr), every point measured in the graph (RESULT by SAMPLE and RESULT by OPERATOR), and the relationship between the operators’ findings (SAMPLE: OPERATOR interaction).

The Gage R&R statistical analysis of the digital measurement technique for analyzing the volume of nasal and maxillary sinus airways after a sinus lift using the lateral window approach technique found that the variability attributable to the digital measurement technique was 3.4% (between the measurements of each operator) of the total variability of the samples. The digital measurement technique used to analyze the volume of nasal and maxillary sinus airways after a sinus lift using the lateral window approach technique is considered replicable and reproducible, as the variability was less than 10% (Figure 6 and Figure 7).

The positions of maxillary sinus ostium were located at the upper-medial surface of the maxillary sinus and away from the filling material used in the maxillary sinus. In addition, the maxillary sinus ostium remained permeable before and after the maxillary sinus lift.

## 4. Discussion

The results of this study refute the null hypothesis (H_0_) that the novel digital measurement method does not provide accurate, replicable, and reproducible volumes of maxillary sinus airways after a sinus lift using the lateral window approach technique.

The results showed a volume reduction in both maxillary sinuses, as well as in the nasal and maxillary sinus airway complex, after a sinus lift using the lateral window approach technique and a novel digital measurement method to quantify the maxillary sinus volume changes with an accurate, replicable, and reproducible approach.

The maxillary sinus is an anatomical structure that requires a 3D radiological study for its accurate assessment; in addition, the accuracy of 3D radiological techniques has been previously demonstrated when compared to conventional 2D radiological techniques (orthopantomography) for measuring the limits of the maxillary sinus and the surrounding anatomical structures [11]. Moreover, the development of digital therapeutic planning software has enabled the volumetric analysis of the maxillary sinus dimensions [8,33]. Schriber et al. analyzed the volumetric changes of the maxillary sinus after tooth extraction using a customized software program, although they found no statistically significant differences (*p* > 0.05) between the volume of the maxillary sinus of dentulous and edentulous patients [34]. Uchida et al. described a procedure for quantifying the volume of grafting material needed to perform a sinus lift using the lateral window approach technique, finding that 0.70 cm^3^ of grafting material was needed to lift the maxillary sinus by 5 mm, 1.92 cm^3^ of grafting material to lift it by 10 mm, 4.02 cm^3^ of grafting material to lift it by 15 mm, and 6.19 cm^3^ of grafting material to lift it by 20 mm [3]. Favato el al. analyzed the survival of dental implants as well as the stability over time of grafting materials after sinus lift, comparing frozen autologous particulate bone, hydroxyapatite, β-tricalcium phosphate, and β-tricalcium phosphate plus Endogain; they found no statistically significant differences (*p* > 0.05) between the stability of the aforementioned grafting materials [35]. Sahlstrand-Johnson et al. analyzed the volumetric dimensions of maxillary and frontal sinuses through computed tomography and Doppler measurements in patients with rhinosinusal pathology [36]. Kreennmair et al. described a procedure for quantifying the volume of grafting material needed to perform a sinus lift using the lateral window approach technique using the predefined dimensions of the pixels of the computed tomography scan sections [37]; however, these measurement techniques are difficult to apply in clinical practice. Therefore, a replicable and reproducible measurement technique must be found to provide replicable and reproducible volumes of the maxillary sinus airways after sinus lifts using the lateral window approach technique. Arias-Irimia et al. used axial tomography scan images and therapeutic planning software (Compunet) to preoperatively quantify bone graft volume [22]. Kirmeier et al. performed measurements using Sienet Magic View to analyze bone graft loss after sinus lift using the lateral window approach technique [38]. Giacommini et al. developed a procedure to automatically quantify the airway volume of the maxillary sinus based on CBCT scan images of patients with rhinosinusitis and septum deviation by using a complex algorithm [39]. Gerken et al. developed a novel computerized technique to quantify the resorption of bone crest and the pneumatization of the maxillary sinus by analyzing 2387 computed tomography scan images in a therapeutic planning software (Materialise) [35]; however, the aforementioned measurement techniques were not confirmed to be replicable and reproducible. The present measurement procedure showed no statistically significant differences between operators (*p* > 0.05), as well as replicability and reproducibility variability of 3.4%. Furthermore, this measurement technique is easily applicable to clinical practice because it only requires a CBCT scan and therapeutic digital planning software, which are becoming increasingly widespread. It could also prove useful in planning the volume of necessary grafting materials, self-assessing sinus lift outcomes, and preventing postoperative complications such as perforation of the Schneiderian membrane (the surgeon’s competence, sinus anatomy, instruments needed for surgery, patient sedation, and surgeon stress levels [40,41] can impact the risk of perforations). It is, therefore, highly recommended that outcomes of sinus lift procedures be analyzed with a view to make the treatment more predictable.

Finally, the clinical interest of the novel digital measurement technique lies in its ability to provide a method to accurately measure the notable decrease in the volume of the maxillary sinus after sinus lift, especially in bilateral sinus lift, using the lateral window approach technique. Some authors have highlighted the clinical relevance of the preoperative evaluation of maxillary sinus lift [28,42]; especially in the lateral window approach technique which present higher sinusitis prevalence (12.1%) than the crestal approach technique (4.1%) [43]. Therefore, an accurate, repeatable, and reproducible measurement technique for the pre-operative assessment of the nasal and maxillary sinuses complex would be useful for accurately planning the surgical treatment approach. In addition, the maxillary sinus lift has been associated to the hypoplasia of the maxillary sinus, which can cause headaches, facial pain, and nasal symptoms [27]. Moreover, the decreases in the volume of the maxillary sinus could lead the development of obstructive sleep apnea, preventing normal breathing during sleep [30]. Therefore, it is recommended that further research analyze the risk of obstructive sleep apnea associated with sinus lift.

Additionally, we analyzed the position and the permeability of the maxillary sinus before and after maxillary sinus lift. If the patency of the maxillary sinus ostium is blocked, clearance of the maxillary sinus can be compromised, increasing the risk of maxillary sinusitis and even obstructive sleep apnea [32]. The maxillary sinus ostium remained permeable before and after the maxillary sinus lifts using the lateral window approach technique due the remote location of the maxillary sinus ostium from the filling material used, showing a reduced risk of sinusitis and obstructive sleep apnea.

## 5. Conclusions

Bearing in mind the limitations of this study, the results indicate that the novel digital method proposed is a replicable, reproducible, and accurate measurement technique for analyzing the volume of nasal and maxillary sinus airways after sinus lifts using the lateral window approach technique, easily applicable to clinical practice.

## Figures and Tables

**Table 1:** Descriptive statistics of the preoperative and postoperative volumes of the right maxillary sinus (mm^3^), left maxillary sinus (mm^3^), and nasal and maxillary sinus airway complex after sinus lift using the lateral window approach technique.

Study Group	*n*	Mean	SD	Minimum	Maximum
Preoperative right maxillary sinus	30	12,032.436	596.232	12,396.129	12,992.188
Postoperative right maxillary sinus	30	9710.983	403.759	9520.837	10,482.038
Right maxillary sinus difference	30	3057.753	287.885	2807.085	3284.989
Preoperative left maxillary sinus	30	19,783.362	1093.027	19,381.378	20,137.153
Postoperative left maxillary sinus	30	15,710.874	603.637	14,284.251	15,137.157
Left maxillary sinus difference	30	4164.266	393.902	3827.255	4402.231
Preoperative nasal and maxillary sinus complex	30	42,665.205	4185.422	37,963.657	51,316.126
Postoperative nasal and maxillary sinus complex	30	36,098.362	7752.174	23,564.275	46,449.285
Nasal and maxillary sinus complex difference	30	6566.105	5604.184	1570.457	17,628.227

SD: standard deviation.

**Table 2:** Descriptive statistics for the two measurements performed by the two operators for the Gage R&R statistical analysis.

Operator	Trial	*n*	Mean	SD	Minimum	Maximum
A	1	2	12,710.500	603.162	12,284.000	13,137.000
	2	2	12,707.000	602.455	12,281.000	13,133.000
B	1	2	12,707.000	601.041	12,282.000	13,132.000
	2	2	12,708.500	601.748	12,283.000	13,134.000

SD: standard deviation.

## Data Availability

Data may be available on request pursuant to relevant restrictions (e.g., privacy and ethical).

## References

[1] Müller F., Naharro M., Carlsson G.E. (2007). What are the prevalence and incidence of tooth loss in the adult and elderly population in Europe?. Clin. Oral Implants Res..

[2] Eufinger H., König S., Eufinger A. (1997). The role of alveolar ridge width in dental implantology. Clin. Oral Investig..

[3] Eufinger H., König S., Eufinger A., Machtens E. (1999). Significance of the height and width of the alveolar ridge in implantology in the edentulous maxilla. Analysis of 95 cadaver jaws and 24 consecutive patients. Mund Kiefer Gesichtschir..

[4] Emtiaz S., Caramês J.M., Pragosa A. (2006). An alternative sinus floor elevation procedure: Trephine osteotomy. Implant. Dent..

[5] Uchida Y., Goto M., Katsuki T., Akiyoshi T. (1998). A cadaveric study of maxillary sinus size as an aid in bone grafting of the maxillary sinus floor. J. Oral Maxillofac. Surg..

[6] Tatum H. (1986). Maxillary and sinus reconstructions. Dent. Clin. North. Am..

[7] Boyne P.J., James R.A. (1980). Grafting of the maxillary sinus floor with autogenous marrow and bone. J. Oral Surg..

[8] Arias-Irimia O., Barona-Dorado C., Martínez-Rodríguez N., Ortega-Aranegui R., Martínez-González J.M. (2010). Pre-operative evaluation of the volume of bone graft in sinus lifts by means of CompuDent. Med. Oral Patol Oral Cir. Bucal..

[9] Pramstraller M., Farina R., Franceschetti G., Pramstraller C., Trombelli L. (2011). Ridge dimensions of the edentulous posterior maxilla: A retrospective analysis of a cohort of 127 patients using computerized tomography data. Clin. Oral Implants Res..

[10] Haas A.J. (1961). Rapid Expansion of the maxillary dental arch and nasal cavity by opening the midpalatal Suture. Angle Orthod..

[11] Warren D.W., Hershey H.G., Turvey T.A., Hinton V.A., Hairfield W.M. (1987). The nasal airway following maxillary expansion. Am. J. Orthod. Dentofac. Orthop.

[12] Krebs A. (1959). Expansion of the Midpalatal Suture, Studied by Means of Metallic Implants. Acta Odontol. Scand..

[13] El H., Palomo J.M. (2010). Measuring the airway in 3 dimensions: A reliability and accuracy study. Am. J. Orthod. Dentofacial Orthop..

[14] Da Silva Filho O.G., Boas M.C., Capelozza Filho L. (1991). Rapid maxillary expansion in the primary and mixed dentitions: A cephalometric evaluation. Am. J. Orthod. Dentofacial Orthop..

[15] Akkaya S., Lorenzon S., Uçem T.T. (1999). A comparison of sagittal and vertical effects between bonded rapid and slow maxillary expansion procedures. Eur. J. Orthod..

[16] Chung C.H., Font B. (2004). Skeletal and dental changes in the sagittal, vertical, and transverse dimensions after rapid palatal expansion. Am. J. Orthod. Dentofacial Orthop..

[17] Montgomery W.M., Vig P.S., Staab E.U., Matteson S.R. (1979). Computed tomography: A three-dimensional study of the nasal airway. Am. J. Orthod..

[18] Hilberg O., Jackson A.C., Swift D.L., Pedersen O.F. (1989). Acoustic rhinometry: Evaluation of nasal cavity geometry by acoustic reflection. J. Appl. Physiol..

[19] Oliveira De Felippe N.L., Da Silveira A.C., Viana G., Kusnoto B., Smith B., Evans C.A. (2008). Relationship between rapid maxillary expansion and nasal cavity size and airway resistance: Short- and long-term effects. Am. J. Orthod. Dentofacial Orthop..

[20] Babacan H., Sokucu O., Doruk C., Ay S. (2006). Rapid maxillary expansion and surgically assisted rapid maxillary expansion effects on nasal volume. Angle Orthod..

[21] Doruk C., Sökücü O., Biçakçi A.A., Yilmaz U., Taş F. (2007). Comparison of nasal volume changes during rapid maxillary expansion using acoustic rhinometry and computed tomography. Eur. J. Orthod..

[22] Al-Rawi N.H., Uthman A.T., Abdulhameed E., Al Nuaimi A.S., Seraj Z. (2019). Concha bullosa, nasal septal deviation, and their impacts on maxillary sinus volume among Emirati people: A cone-beam computed tomography study. Imaging Sci. Dent..

[23] Lotfi V., Ghoneima A., Lagravere M., Kula K., Stewart K. (2018). Three-dimensional evaluation of airway volume changes in two expansion activation protocols. Int. Orthod..

[24] Rômulo de Medeiros J., Ferraro Bezerra M., Gurgel Costa F.W., Pinheiro Bezerra T., de Araújo Alencar C.R., Studart Soares E.C. (2017). Does pterygomaxillary disjunction in surgically assisted rapid maxillary expansion influence upper airway volume? A prospective study using Dolphin Imaging 3D. Int. J. Oral Maxillofac. Surg..

[25] Wake M., Shankar L., Hawke M., Takeno S. (1993). Maxillary sinus hypoplasia, embryology, and radiology. Arch. Otolaryngol. Head Neck Surg..

[26] Sánchez Fernández J.M., Anta Escuredo J.A., Sánchez Del Rey A., Santaolalla Montoya F. (2000). Morphometric study of the paranasal sinuses in normal and pathological conditions. Acta Otolaryngol..

[27] Bhargava A., Khanduri S., Shakeel M., Srivastava S., Varshney P. (2016). Maxillary sinus hypoplasia—a not-so-uncommon clinical entity: A review. Clin. Rhinol..

[28] Alsufyani N., El-Hakim H., Major P. (2021). Prevalence of maxillary sinus hypoplasia and association with variations in the sinonasal complex: A cone beam CT study. Clin. Oral Investig..

[29] Karslioglu H., Sumer A.P. (2020). Evaluation of maxillary sinus findings after dental implant and sinus floor augmentation procedures with cone-beam computed tomography. Niger J. Clin. Pract..

[30] Rowley J.A., Lareau S., Fahy B.F., Garvey C., Sockrider M. (2017). What Is Obstructive Sleep Apnea in Adults?. Am. J. Respir. Crit. Care Med..

[31] Kim Y.J., Shin H.K., Lee D.Y., Ryu J.J., Kim T.H. (2020). Decreased maxillary sinus volume is a potential predictor of obstructive sleep apnea. Angle Orthod..

[32] Lee J.W., Yoo J.Y., Paek S.J., Park W.J., Choi E.J., Choi M.G., Kwon K.H. (2016). Correlations between anatomic variations of maxillary sinus ostium and postoperative complication after sinus lifting. J. Korean Assoc. Oral Maxillofac. Surg..

[33] Baciut M., Hedesiu M., Bran S., Jacobs R., Nackaerts O., Baciut G. (2013). Pre- and postoperative assessment of sinus grafting procedures using cone-beam computed tomography compared with panoramic radiographs. Clin. Oral Impl. Res..

[34] Schriber M., Bornstein M.M., Suter V.G.A. (2019). Is the pneumatisation of the maxillary sinus following tooth loss a reality? A retrospective analysis using cone beam computed tomography and a customised software program. Clin. Oral Investig..

[35] Favato M.N., Vidigal B.C., Cosso M.G., Manzi F.R., Shibli J.A., Zenóbio E.G. (2015). Impact of human maxillary sinus volume on grafts dimensional changes used in maxillary sinus augmentation: A multislice tomographic study. Clin. Oral Implants Res..

[36] Sahlstrand-Johnson P., Jannert M., Strömbeck A., Abul-Kasim K. (2011). Computed tomography measurements of different dimensions of maxillary and frontal sinuses. BMC Med. Imaging.

[37] Krennmair G., Krainhöfner M., Maier H., Weinländer M., Piehslinger E. (2006). Computerized tomography-assisted calculation of sinus augmentation volume. Int. J. Oral Maxillofac. Implants..

[38] Kirmeier R., Arnetzl C., Robl T., Payer M., Lorenzoni M., Jakse N. (2011). Reproducibility of volumetric measurements on maxillary sinuses. Int. J. Oral Maxillofac. Surg..

[39] Giacomini G., Pavan A.L.M., Altemani J.M.C., Duarte S.B., Fortaleza C.M.C.B., Miranda J.R.A., de Pina D.R. (2018). Computed tomography-based volumetric tool for standardized measurement of the maxillary sinus. PLoS ONE.

[40] Juzikis E., Gaubys A., Rusilas H. (2018). Uses of maxillary sinus lateral wall bony window in an open window sinus lift procedure: Literature review. Stomatologija.

[41] Testori T., Weinstein T., Taschieri S., Wallace S.S. (2019). Risk factors in lateral window sinus elevation surgery. Periodontology 2000.

[42] Chen Y.W., Lee F.Y., Chang P.H., Huang C.C., Fu C.H., Huang C.C., Lee T.J. (2018). A paradigm for evaluation and management of the maxillary sinus before dental implantation. Laryngoscope.

[43] Kim Y.K., Hwang J.Y., Yun P.Y. (2013). Relationship between prognosis of dental implants and maxillary sinusitis associated with the sinus elevation procedure. Int. J. Oral Maxillofac. Implant..

